# Identification of urinary volatile organic compounds as a potential non-invasive biomarker for esophageal cancer

**DOI:** 10.1038/s41598-023-45989-1

**Published:** 2023-10-30

**Authors:** Qi Liu, Shuhai Li, Yaping Li, Longchen Yu, Yuxiao Zhao, Zhihong Wu, Yingjing Fan, Xinyang Li, Yifeng Wang, Xin Zhang, Yi Zhang

**Affiliations:** 1 Department of Clinical Laboratory, Qilu Hospital of Shandong University, 107 Wenhua Xi Road, Jinan, 250012 Shandong China; 2 Shandong Engineering Research Center of Biomarker and Artificial Intelligence Application, 107 Wenhua Xi Road, Jinan, 250012 Shandong China; 3 Department of Thoracic Surgery, Qilu Hospital of Shandong University, 107 Wenhua Xi Road, Jinan, 250012 Shandong China; 4 Department of Traditional Chinese Medicine, 107 Wenhua Xi Road, Jinan, 250012 Shandong China

**Keywords:** Biochemistry, Cancer, Oncology

## Abstract

Early diagnosis of esophageal cancer (EC) is extremely challenging. The study presented herein aimed to assess whether urinary volatile organic compounds (VOCs) may be emerging diagnostic biomarkers for EC. Urine samples were collected from EC patients and healthy controls (HCs). Gas chromatography-ion mobility spectrometry (GC-IMS) was next utilised for volatile organic compound detection and predictive models were constructed using machine learning algorithms. ROC curve analysis indicated that an 8-VOCs based machine learning model could aid the diagnosis of EC, with the Random Forests having a maximum AUC of 0.874 and sensitivities and specificities of 84.2% and 90.6%, respectively. Urine VOC analysis aids in the diagnosis of EC.

## Introduction

Esophageal cancer (EC) is a malignant tumor originating from the epithelium of the esophagus. EC is mainly classified into squamous cell carcinoma and adenocarcinoma dependent on the type of pathology^1^. In 2020, there were an estimated 604,000 new cases and 544,000 deaths associated with EC worldwide^2^. In China, EC has the sixth highest incidence rate and the fifth highest mortality rate^3^. Although there has been a steady reduction in incidence in recent years, the overall disease burden is still high due to the high risk of EC to human health. According to statistics, over 90% of the Chinese population has squamous epithelial carcinoma, making it difficult to draw conclusions from research findings on adenocarcinoma in Western countries. In the early stages of its development, EC mainly presents as a choking sensation when swallowing food, a foreign body sensation, or difficulty in swallowing. As such, EC is often overlooked. Currently, the treatment of EC is a combination of surgery, radiotherapy and chemotherapy, but due to late diagnosis, most patients cannot undergo surgical excision^4^. Tumor markers play a significant role in monitoring and treating tumors. However, common biomarkers, such as CEA, CA 19–9 and CA 125, have low sensitivity and specificity^5–7^. Moreover, false positives can have bee reported due to nonspecific elevations in distinct digestive tract disorders^8–11^. The described issues often result in a poor prognosis for EC, with a 5-year survival rate of only 15–25%^12^. Thus, developing new biomarkers for EC represent an urgent unmet medical need.

Volatile organic compounds (VOCs) are an important component of human metabolites and can be detected by headspace analysis. As the occurrence of VOCs is primarily associated with oxidative stress, inflammation and changes in cellular metabolism, VOCs are considered to be a systemic and local biomarker that can provide unique information about ongoing biochemical processes and thus an individual’s health status^13–15^. Exploring changes in human VOCs has been reported to be useful in the diagnosis of tumors. VOCs detected in urine can be used in combination to construct an effective diagnostic model^16–22^ (Supplementary Table S1). Similarly, studies investigating diagnostic models based on VOCs detected in exhaled breath have been reported in lung cancer^23^. VOCs in bile and other samples have further been demonstrated to be useful for the diagnosis of tumors^24^.

The aim of the present study was to identify differential VOCs in patients with EC compared to healthy controls (HCs) and to subsequently develop a volatile biomarker model that could aid in the early diagnosis of EC. To our knowledge, we have revealed for the first time that VOC levels detected by using Gas chromatography-ion mobility spectrometry (GC-IMS) can be utilised as noninvasive biomarkers for the diagnosis of EC.

## Results

### Participant characteristics

**Figure 1:**
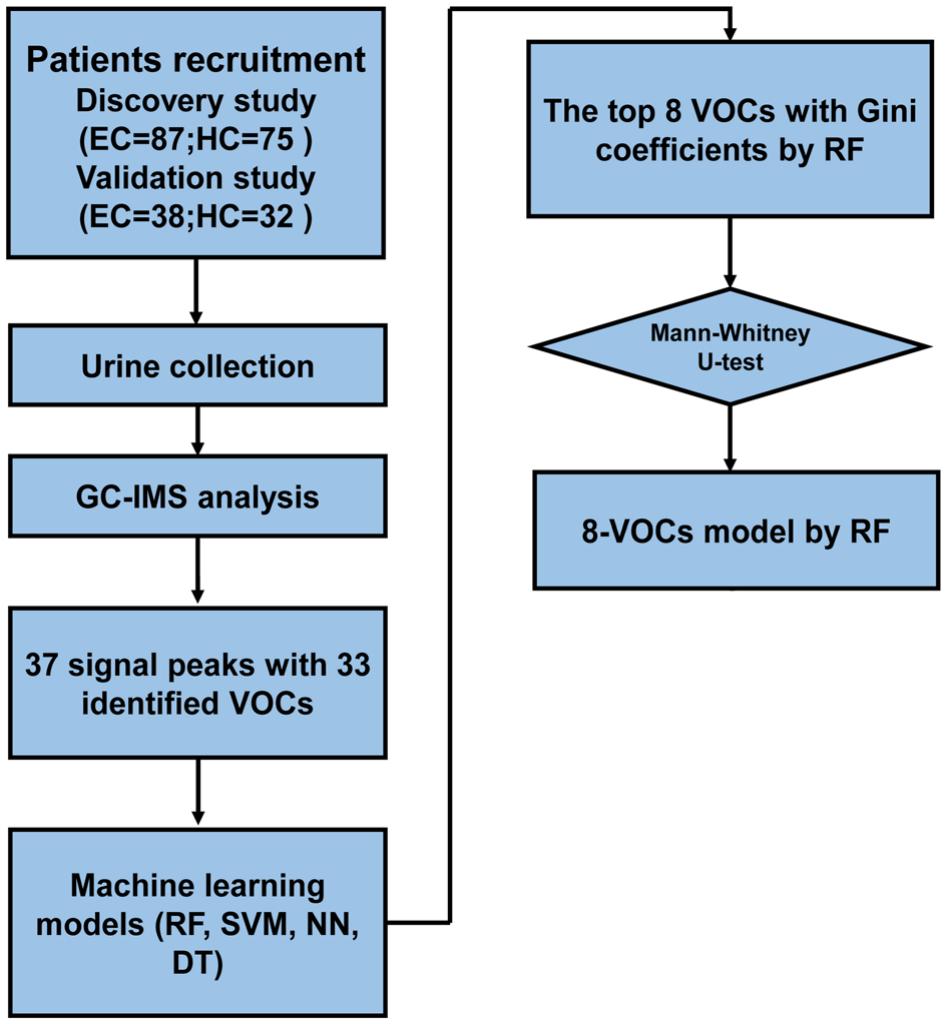
Flowchart of study design.

The overall study design is shown in Fig. 1 In the discovery study, the headspace outputs from 241 urine samples were analyzed after 23 patients were excluded prior to surgery. Another 19 patients were excluded after post-operative pathological confirmation. In addition, 37 HCs were excluded; the specific reasons for these exclusions are shown in Supplementary Fig. S1A. Finally, sample data from 162 (EC = 87, HC = 75) patients were included in the analysis for collation (Table 1). Similarly, in the validation study (Supplementary Fig. S1B), the headspace outputs of 125 urine samples were initially analyzed. Nineteen patients were excluded before the surgery and an additional 14 patients were excluded after postoperative pathological confirmation. Furthermore, 22 HCs were excluded. Finally, data from 70 patient samples (EC = 38, HC = 32) were included in the analysis (Table 1). The final analyzed cohort is illustrated in Supplementary Fig. S1C.

**Table 1:** Patient characteristics.

Characteristics	EC (*N* = 125)		HC (*N* = 107)	
	Discovery	Validation	Discovery	Validation
Cases	87	38	75	32
Demographic data				
Age, years, means ± SD	63.9 ± 7.4	65.1 ± 8.9	57.2 ± 9.1	57.1 ± 7.6
Gender (male)	75 (86.2)	33 (86.8)	64 (85.3)	22 (68.8)
Body height, cm, means ± SD	168.3 ± 6.7	168.1 ± 7.2	170.3 ± 8.4	171.4 ± 8.2
Weight, means ± SD	67.7 ± 12.4	65.7 ± 9.1	63.5 ± 5.3	62.5 ± 6.4
BMI, medians (IQR)	23.2 (21.1–26.2)	22.9 (21.7–24.3)	22.1 (20.7–22.8)	21.4 (20.5–22.0)
Smoking history	63 (72.4)	24 (63.2)	18 (24.0)	5 (15.6)
Current smoking	43 (49.4)	11 (28.9)	14 (18.7)	2 (6.3)
Alcohol history	57 (65.5)	27 (71.1)	16 (21.3)	4 (12.5)
Current alcohol drinking	39 (44.8)	15 (39.5)	12 (16.0)	3 (9.4)
Comorbidities				
Diabetes	7 (8.0)	4 (10.5)	7 (9.3)	2 (6.3)
Cardiovascular disease	5 (5.7)	3 (7.9)	6 (8.0)	3 (9.4)
Respiratory disease	2 (2.3)	0	2 (2.7)	0
Pathological data				
Cancer type				
SC/AC	84/3 (96.6/3.4)	35/3 (92.1/7.9)		
Pathological stage				
I/II/III/IV	18/27/29/13	6/12/16/4		
	(20.7/31.1/33.3/14.9)	(15.8/31.6/42.1/10.5)		
Vascular embolus or neural invasion	46 (52.9)	16 (42.1)		
Lymph node metastasis	43 (49.4)	21 (55.3)		
Differentiation degree				
G1	22 (25.3)	7 (18.4)		
G2	28 (32.2)	11 (28.9)		
G3	37 (42.5)	20 (52.6)		

Data are the mean ± standard deviation or number (%).

*BMI* body mass index, *IQR* interquartile range, *SC* squamous carcinoma, *AC* adenocarcinoma, *N* number.

### VOC profile analysis in EC and HC patients

**Figure 2:**
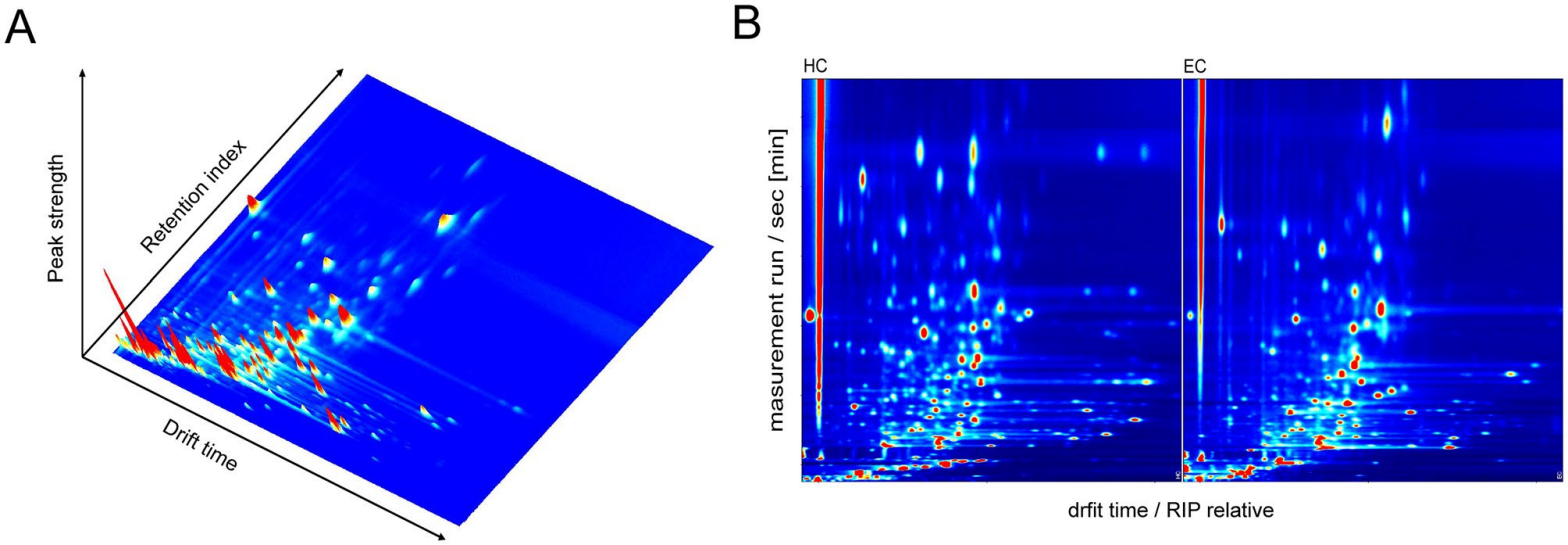
Urine VOCs profile detected in EC and HC. (**A**) 3D spectral map of VOC generated by GC-IMS. (**B**) A 2D map showed the difference in the VOCs when comparing an EC sample and an HC sample so that the drift time and retention index of different VOCs can be intuitively observed.

Similar to our previous studies, substances were characterized by the retention index of the molecule and the drift time of its ions before being quantified by the intensity of the signal peaks^24^. Therefore, for each sample to be measured, three-dimensional data (retention index, drift time, peak strength) was obtained (Fig. 2A). The data was analyzed by comparing the mean of a 2D spectrogram (top view of a 3D spectrogram, with colors indicating peak intensity), where each “dot” (Fig. 2B) was a signal peak. The 2D coordinates of the signal peak location were retrieved (retention index × drift time) to characterize the compound, and the integration region was boxed to integrate the signal peak to obtain the peak height. Thirty-seven VOC peaks were selected based on retention indices and drift times (Supplementary Table S2). See Supplementary Table S3 for specific peak height values.

### Diagnostic performance of urine VOCs with machine learning algorithms

We used a machine learning approach to analyze heterogeneous VOC signals in EC patients and HCs. Combined with the peak height of the above VOCs, four popular machine learning algorithms (random forests (RF), neural network (NN), support vector machines (SVM), decision trees (DT)) were used to construct diagnostic models. Table 2 demonstrates the results of receiver operating characteristic (ROC) analysis of the four models from the urine validation study. The RF model had the highest area under the curve (AUC) of 0.863, with a sensitivity of 78.9% and a specificity of 93.8%. Note: The F1 value is the reconciled mean of the check accuracy and the check completeness rates, and measures the balance of precision and recall of the model. F1 = (2Precision × Recall)/(Precision + Recall).

**Table 2:** Diagnostic performance of VOCs with machine learning algorithm.

Model	Accuracy	Precision	Recall	F1	Specificity	AUC
RF	0.857	0.938	0.789	0.857	0.938	0.863
NN	0.786	0.829	0.763	0.795	0.812	0.788
SVM	0.786	0.848	0.737	0.789	0.844	0.790
DT	0.829	0.906	0.763	0.829	0.906	0.835

Recall is equivalent to sensitivity.

*RF* random forests, *NN* neural network, *SVM* support vector machines, *DT* decision trees, *AUC* area under the curve.

### Estimation of importance of the urinary VOCs using Random forests analysis

**Figure 3:**
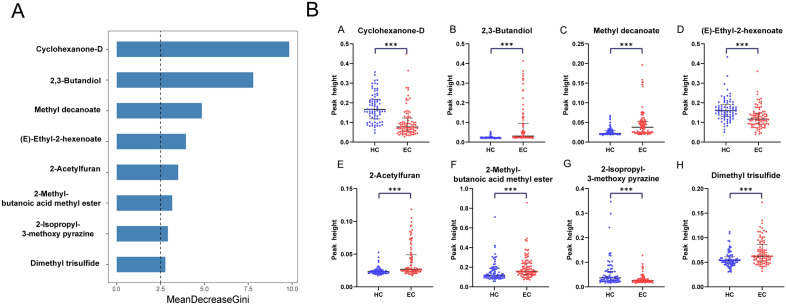
Estimation of importance of the urinary VOCs using Random forests analysis. (**A**) The top eight VOCs with Gini coefficients by RF. (**B**) Comparisons of peak height of volatile organic compounds in patients with EC and HC. The peak height of Cyclohexanone-D (A), 2,3-Butandiol (B), Methyl decanoate (**C**), (E)-Ethyl-2-hexenoate (**D**), 2-Acetylfuran (**E**), 2-Methyl-butanoic acid methyl ester (**F**), 2-Isopropyl-3-methoxy pyrazine (**G**), Dimethyl trisulfide (**H**). *P < 0.05, **P < 0.01, ***P < 0.001 (Mann–Whitney U-test). The error bars in scatter plots represent the median and interquartile range.

**Figure 4:**
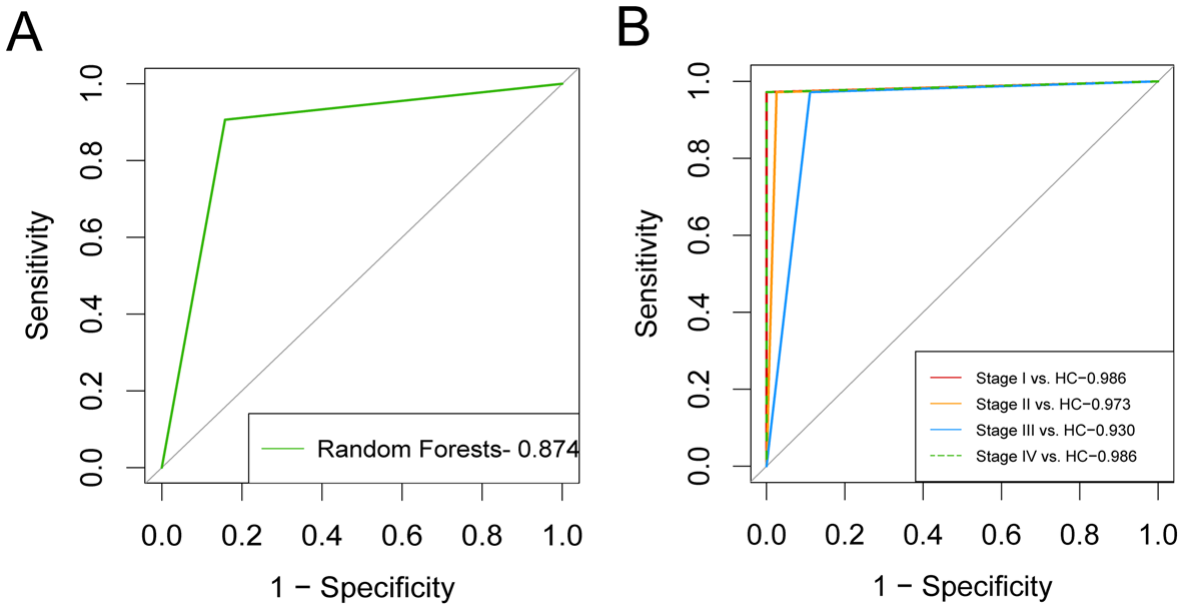
ROC curves analysis for RF machine learning model. (**A**) The AUC area of 8-VOCs model by RF in validation study. (**B**) Identification of HCs and stage I-IV EC using 8-VOCs model.

The results in Table 2 are obtained from the four machine learning models based on the analysis of all 37 VOCs. The RF algorithm showed the most superior results, so we used RF for the final model construction in the subsequent analysis. Through RF model analysis, the top eight VOCs with Gini coefficients were further analyzed (Fig. 3A). Detailed importance ranking of 37 VOCs are shown in Supplementary Fig. S2. Compared to HCs, five that were up-regulated (2,3-Butandiol, 2-Acetylfuran, Dimethyl trisulfide, 2-Methyl-butanoic acid methyl ester, Methyl decanoate) and three that were down-regulated ((E)-Ethyl-2-hexenoate, 2-Isopropyl-3-methoxy pyrazine, Cyclohexanone-D) (Fig. 3B). In combination with these eight VOCs, we used RF to build a new diagnostic model. The AUC area of 8-VOCs model reached 0.874 (Fig. 4A). In addition, HCs and four EC staging groups were compared using the 8-VOCs model, a diagnostic model that can well distinguish the different staging groups from controls (Fig. 4B and Supplementary Table S4).

### Urine biomarkers and pathological parameters

As shown in Table 1, in the discovery study, the majority of patients exhibited squamous carcinomas (96.6%) while a minority presented with adenocarcinomas (3.4%). The proportions were 51.8% and 48.2% for early (I + II) and advanced (III + IV) stages, respectively. As clinical stage plays an extremely important role in the prognosis of patients with EC, we compared the levels of these eight VOCs in controls and patients with different stages. Controls had higher levels of (E)-Ethyl-2-hexenoate, 2-Isopropyl-3-methoxy pyrazine and lower levels of 2-Acetylfuran when compared to stage I, II, III EC patients. 2-Methyl-butanoic acid methyl ester was significantly higher in stages III and IV than in controls, whereas no significant differences were observed in stages I and II (Supplementary Table S5). Regarding comparisons of VOCs among different cancer stages (Supplementary Fig. S3), 2-Isopropyl-3-methoxy pyrazine provided a significantly higher peak height in patients at stage IV, but the difference in peak height between stages for the other VOCs was not statistically significant.

### Correlation analysis of VOCs in urine

Correlation analyses demonstrated a significant differential enrichment of metabolically generated VOCs in urine when comparing levels in the EC and HC populations (Supplementary Fig. S4). These data suggest a different pattern of VOC associations between the two groups.

## Discussion

In the present study, we explored the measurement of urinary VOCs for the early diagnosis of EC and subsequent data analysis resulted in several meaningful findings. Firstly, GC-IMS was efficient in differentiating between patients with EC and healthy subjects by the detection of VOCs. Secondly, a diagnostic model was constructed by machine learning to clarify the diagnostic value of VOCs in urine. Thirdly, eight different VOCs were identified in urine that may play a relevant role in the diagnosis of EC.

Although research regarding VOCs in EC metabolites has been ongoing for many years, there are currently no biomarkers for VOCs that can be utilized in clinical screening. Previous studies of VOCs in human metabolites in patients diagnosed with EC versus non-cancerous subjects have differed in many aspects^25–29^. Broadly, all relevant studies have achieved a diagnostic accuracy of over 80%, and strikingly, a diagnostic model AUC area of 0.97 was achieved in a study exploring exhaled breath VOCs in EC. The primary categories of differential VOCs reported so far are aldehydes, ketones, acids and phenols, however, differences in the specific compound categories remains an issue. The platforms most frequently used in previous studies were selected ion flow tube mass spectrometry (SIFT-MS) and proton transfer reaction mass spectrometer (PTR-MS). In this regard, an innovative method employing GC-IMS assays in our study led to similar results. Previously, teams have used GC-IMS to detect and characterise differences in VOCs between pancreatic, colorectal and liver cancer patients and their non-cancer control populations^16,30,31^. Of course, variation in results is present when comparing different metabolic platforms, sample types, and methodological procedures. These aspects will require refinement in the future by more extensive clinical studies in which sufficient sample data are collected, and by the development of standardized sample handling protocols.

A variety of metabolite VOCs have been extensively studied in previous reports. Exhaled breath is one of the earlier body fluid samples to be used for VOCs detection due to its ease of collection and other advantages. Kumar et al. clearly differentiated EC from patients with benign gastrointestinal disorders based on exhaled gas VOCs^27,32^. In addition, through a multicentre validation study, Markar et al. constructed a diagnostic model for 5-VOCs based on the composition of butyric acid, valeric acid, hexanoic acid, butyraldehyde, and decanal by analysing exhaled breath samples from esophagogastric cancer patients and control patients using SIFT-MS^28^. The exploration of urinary VOCs has mainly focused on urological tumors such as prostate cancer and bladder cancer. Gao et al. constructed an 11-VOCs model for differentiation between prostate cancer and non-cancer patients based on their urinary VOCs by GC–MS assay. Gao et al. constructed an 11-VOCs model for differentiation between prostate cancer and non-cancer patients based on their urinary VOCs by GC–MS assay^17^. Similarly, Tyagi's team examined urinary VOCs in patients with prostate and bladder cancer and, in addition to finding differences between the tumor and control groups, the VOCs were similarly different between patients with the two types of tumors^22^. There are also studies on bile, faeces and other VOCs, which have established new methods for the diagnosis of cholangiocarcinoma, pancreatic cancer and colorectal cancer, respectively^33–36^.

Unlike in previous studies, we first screened for potential diagnostic VOCs based on differences identified between the urine of cancer patients and of healthy patients. Four diagnostic models were used for the analysis. To avoid overfitting of the model, the feasibility of the model was subsequently confirmed by external validation. Afterwards, we selected the RF with the best performance as the diagnostic model for analysis, and finally selected eight VOCs for model construction. The effects of patient diet, medication and serious complications were overcome by stratification and exclusion criteria during the sample collection process. The effects of air exposure were limited by reducing the time taken for samples to travel from the ward to the laboratory. Storage times prior to final VOC testing were kept consistent to avoid differences in long-term storage loss at low temperatures. Distinct to previous studies, herein we have collated a urine metabolomics database consisting of 125 patients with EC and 107 HCs. To our knowledge, we have obtained the largest number of samples for the study of urinary VOCs in patients with EC.

Machine learning has been widely used in the medical field, and several medical-related algorithms are briefly described below^37^. Random forest is an idea of integrated learning, where data obtained by random sampling is fed into numerous weak learners and voted on to obtain the final output. Neural Networks, also known as Artificial Neural Networks, are algorithms that attempt to mimic the concepts of how the human brain interprets and draws conclusions from information using multiple layers of computation. The learning process of NNs can be supervised or unsupervised, and are essentially mathematical models designed to deal with complex and disparate information. Support Vector Machine is a supervised learning algorithm widely used in classification and regression tasks. The core idea of SVM is to find a hyperplane that maximises the spacing between two classes to achieve good classification. The decision tree algorithm is a method for approximating the value of a discrete function. It is a typical classification method, where the data is first processed, an inductive algorithm is used to generate readable rules and a decision tree, and then the new data is analysed using the decisions. Essentially decision tree is the process of classifying data by a set of rules.

Endogenous VOCs are primarily produced by human cells in the process of energy metabolism and oxidative stress. VOCs eventually reach alveolar cells, renal epithelial cells and intestinal epithelial cells via the blood to be excreted, and as such they directly reflect changes in human metabolism. Many studies have confirmed the validity of analysis of VOC in exhaled breath for tumor diagnosis^38,39^. However, VOCs in exhaled breath are easily influenced by other airborne substances, leading to confounding of analysis. The choice of urine as study samples is likely to avoid interference from external factors and to yield reliable conclusions.

For the known VOCs detected, we were able to explore their relationship with tumors. The model constructed on the basis of the eight VOCs could distinguish them well in the urine of patients when comparing between the early and late stages of tumor development. These findings may suggest that certain VOCs are associated with the proliferative migration of tumors.

Of the VOCs associated with EC discovered in this study, some have been demonstrated to have potential as diagnostic biomarkers for other diseases. For instance, 2-Acetylfuran was significantly reduced in plasma extracellular vesicles in Severe acute pancreatitis (SAP) patients^40^, and dimethyl trisulfide in exhaled breath and 4-Heptanone in urine samples are significantly different from breast cancer patients than from non-cancer patients^41,42^. Moreover, 2-Butanone has the potential to be a biomarker for tumors associated with breast^43^ and hepatocellular carcinoma^30^ cancers. In addition, levels of 1-Octen-3-ol and (E)-2-nonenal have been shown to be significantly altered in saliva samples from oral cancer patients compared to the non-cancer patient group^44^. Meanwhile, cyclohexanone has been shown to be differentially expressed in bile samples from gallbladder cancer patients^24^ and in exhaled breath samples from breast^45^ and colorectal cancer patients^46^.

There is a growing interest in investigation of metabolite VOCs in oncology patients. Metabolites such as: urine, bile, exhaled breath, serum and feces have been shown to have potential diagnostic value as tumor identifiers. Unfortunately, research on the mechanisms of production of metabolite VOCs is extremely limited. Metabolic regulation plays a key role in cancer adaptation to oxidative stress^47^, and tumor cells have been reported to promote cancer development and progression through the readjustment of metabolic processes. The relationship between VOCs in urine and oxidative stress has been demonstrated^15^. As such, dimethyl trisulfide has been reported to be associated with oxidative stress^48^. For substance-specific changes, tumor cells are usually accompanied by elevated aldehyde dehydrogenase (ALDH) activity^49^, and ALDHs are known to catalyze the oxidation of exogenous and endogenous aldehyde substrates to their corresponding carboxylic acids^50^. These findings explain the decrease in aldehyde levels in the EC patients in our study. Moreover, changes in ketone levels in metabolites may be associated with altered lipid metabolism in tumor cells^51^.

Currently, GC-IMS is not widely used for the detection of metabolomic markers and as such, the differential VOCs we identified in this study may not be reproducible by other detection platforms. Those platforms for detecting oncology VOCs, such as Gas chromatography-mass spectrometry (GC–MS), SIFT-MS, electronic-nose (eNose), etc. do have different advantages and disadvantages. For example, GC–MS can only be used for the separation and identification of low molecular weight (approx. 50–600 Da) and volatile compounds^52^. For the detection of polar, non-thermal and non-volatile metabolites, the use of chemical derivatization is required prior to analysis. The derivatization reaction utilised to produce volatile compounds increases the sensitivity and accuracy of the assay, enabling efficient analysis of hundreds of compounds^53^. Moreover, SIFT-MS facilitates real-time measurements, and while the instrument is less expensive to maintain and does not require a specialist to operate, it does separate fewer VOCs and consequently provides less information about VOC components^54^. The eNose assay is simple to operate, faster and cheaper, but is susceptible to interference from environmental factors and cannot separate single VOCs components^55^. Compared to the above detection platforms, GC-IMS not only has a high ability to separate complex components, but the ultra-sensitivity of ion mobility spectrometry allows it to detect very small concentrations of VOCs, while the samples do not need to be enriched and concentrated, maintaining their true flavour and making it suitable for rapid detection of large numbers of samples. In addition, the detection time of GC-IMS is considerably shorter and the accuracy of the results is significantly improved compared to other methods^56^.

Metabolomic testing in urine has proven to be an effective method for identifying biomarkers in EC. In the study presented herein, GC-IMS was employed to identify 8 urinary VOCs associated with EC that could accurately distinguish patients with EC from healthy individuals. This study provides an experimental basis for the application of VOC analysis in EC, allowing it to be used in the diagnosis of EC, which has extremely promising clinical applications.

## Limitations of the study

The limitation of this study is that the metabolic pathways of some metabolites in the results have not been identified. Meanwhile, the metabolic relationship between the screened markers and esophageal cancer and their future value for clinical diagnosis still need to be further explored. In addition, the cohort size of the study needs to be further expanded and multi-centre validation needs to be added.

## Methods

### Study population

In the discovery study, a total of 162 patients, including 87 EC patients and 75 HCs, were enrolled in Qilu Hospital of Shandong University from October 2021 to June 2022. Urine samples were collected from all patients before they were tested using GC-IMS to identify candidate VOCs prior to the construction of diagnostic models. The validation cohort consisted of 38 EC patients and 32 HCs enrolled at Qilu Hospital of Shandong University to evaluate the diagnostic model. Inclusion criteria were set as follows: (1) No patients had a history of malignancy, previous radiotherapy or other treatment. (2) Patients could provide fresh urine samples and complete medical records. (3) Patients had undergone radical resection and were pathologically examined for EC. Simultaneously, healthy individuals took physical examinations in our hospital and were selected to serve as the HC group. All individuals in the control group had normal urine, liver and kidney function indicators. No tumors or other major diseases were identified in individuals in the HC group. The study was carried out in accordance with the Declaration of Helsinki. The Ethics Committee of Shandong University's Qilu Hospital approved this study, and each participant signed an informed consent form.

Ultimately, 162 urine samples were obtained for the discovery study (EC = 87, HC = 75), while 70 urine samples were collected for the validation study (EC = 38, HC = 32).

### Sample preparation

The standards were purchased from Sinopharm Chemical Reagent Co., Ltd. for characterisation (including esters, acids, ketones, aldehydes, alkanes, ethers, alcohols, aromatic compounds and other compounds); the chromatographic column model was MXT-WAX, 15 m long, 0.53 mm ID, 1um film thickness, purchased from Restek, USA; and the GC-IMS equipment was purchased from GAS, Dortmund, Germany; High purity nitrogen, purity 99.999%.

Fasting urine samples were obtained from all subjects. Urine samples were collected in standard universal sterile specimen containers and frozen at -80 °C within 3 h. No chemicals were added to the urine prior to freezing. The urine was thawed in a laboratory refrigerator at 4 °C prior to analysis and 2 ml was added to a 20 mL glass sample bottle with a pressurised cap.

### Analysis of the VOCs

VOCs were measured using GC-IMS (“FlavorSpec” brand, Dortmund, Germany). GC-IMS pre-separates complex VOC fractions in urine by GC, which is subsequently tandem with IMS, and then achieves secondary separation based on the mass of the ion to be measured and the one-dimensional collision cross-sectional area. Two-dimensional characterisation can be performed based on retention indices from GC and drift times from IMS, and quantification based on signal response intensity. For GC columns we use the strongly polarised column MXT-WAX. All samples were treated homogenously. Orthogonal experiments were performed to explore the experimental parameters: the optimal sample volume was derived to be 2 mL, incubated at 100 °C for 5 min, and injected with 1000 μL. Firstly, 2 mL of urine was placed in each headspace vial and incubated at 100 °C for 5 min. Subsequently, 1000 μL of gas was extracted from the headspace vial for analysis. Nitrogen was used as the carrier gas. IMS drift gas was maintained at 150 mL/min with the following carrier gas gradients: 0 min: 2 mL/min; 1 min: 2 mL/min; 8 min: 100 mL/min; 10 min: 150 mL/min; 15 min: 150 mL/min. Additional primary parameters were as follows: T1 drift tube temperature: 45 °C; T2 gas chromatography column temperature: 80 °C; T3 inlet temperature: 80 °C; T4 connection line 1: 80 °C and T5 connection line 2: 45 °C.

### Statistical analysis

The GC retention indices and IMS relative drift times were cross-characterised from the NIST database and the drift time data from the self-built IMS database. The peak positions of the compounds were confirmed by comparison with the peak positions of the standards and the retention indices and drift times of the compounds to be tested had to be consistent with the data of the standards at the same time. All compounds were named from the purchased NIST library of spectra.

Continuous data were analyzed using the Mann–Whitney U-test. The R programme (× 64 4.2.0) and the “corrplot” package was used for correlation analysis. The Mann–Whitney U test was used to compare the levels of each VOC. The AUC was calculated on the ROC curves using MedCalc 9.3.9.0. A tenfold cross-validation was used in building the four diagnostic models and was used to select the optimal model parameters. Four machine learning models (RF, NN, SVM, DT) were applied to model construction. RF algorithm evaluated by Gini coefficient. NN uses the stochastic gradient descent (SGD) optimisation algorithm. SVM uses a nonlinear kernel function. The C4.5 algorithm was used for the DT. The error bars in scatter plots represent the median and interquartile range.

### Ethics declarations

The studies involving human participants were reviewed and approved by the Ethics Committee of Qilu Hospital of Shandong University. The study was carried out in accordance with the Declaration of Helsinki. The patients/participants provided their written informed consent to participate in this study.

### Supplementary Information

Supplementary Information.

## Data Availability

The data that support the findings of this study are available from the corresponding author upon reasonable request.
