# Supplementary material for: Identification of urinary volatile organic compounds as a potential non-invasive biomarker for esophageal cancer
Source: Sci Rep. 2023 Oct 30;13:18587. doi: 10.1038/s41598-023-45989-1 (PMC10616168; doi:10.1038/s41598-023-45989-1)
Supplement: Supplementary file 1 — Supplementary Information. [file 41598_2023_45989_MOESM1_ESM.zip › Supplementary files/Supplementary figure legends.docx]

**Identification of Urinary** **Volatile Organic Compounds as a Potential Non-invasive Biomarker for Esophageal Cancer**

**Qi Liu^1,2#^, Shuhai Li^3#^, Yaping Li^1,2^, Longchen Yu^1,2^, Yuxiao Zhao^1,2^, Zhihong Wu^4*^, Yingjing Fan^1,2^, Xinyang Li^1,2^, Yifeng Wang^1,2^, Xin Zhang^1,2^, Yi Zhang^1,2*^**

**Authors’ Afﬁliations:**

^1^Department of Clinical Laboratory, Qilu Hospital of Shandong University, 107 Wenhua Xi Road, Jinan, Shandong 250012, China

^2^Shandong Engineering Research Center of Biomarker and Artificial Intelligence Application, 107 Wenhua Xi Road, Jinan, Shandong 250012, China

^3^Department of Thoracic Surgery, Qilu Hospital of Shandong University, 107 Wenhua Xi Road, Jinan, Shandong 250012, China

^4^Department of Traditional Chinese Medicine, 107 Wenhua Xi Road, Jinan, Shandong 250012, China

^#^These authors contributed equally to this work.

*Corresponding author.

E-mail address: [yizhang@sdu.edu.cn](mailto:yizhang@sdu.edu.cn) (Y.Zhang), 1205134451@qq.com (Z.Wu)

**Supplementary Figure S1. Flowchart of participant recruitment.** The discovery study (A), validation study (B), and final analyzed cohort (C) are shown.

**Supplementary Figure S2. The 37 VOCs with Gini coefficients by RF.**

**Supplementary Figure S3. Comparisons of peak height of VOCs among EC with different pathological stage.** The Kruskal-Wallis tests demonstrated no significant difference in all comparisons (all *P* >0.05) except 2-Isopropyl-3-methoxy pyrazine .

**Supplementary Figure S4. Correlation analysis of VOCs in HCs and patients with EC.** Correlation analysis of Urine VOCs in HC (A) and patients with EC (B).
