# Supplementary material for: Identification of urinary volatile organic compounds as a potential non-invasive biomarker for esophageal cancer
Source: Sci Rep. 2023 Oct 30;13:18587. doi: 10.1038/s41598-023-45989-1 (PMC10616168; doi:10.1038/s41598-023-45989-1)
Supplement: Supplementary file 1 — Supplementary Information. [file 41598_2023_45989_MOESM1_ESM.zip › Supplementary files/Supplementary Table S1.docx]

**Supplementary Table S1. VOCs detected in urine.**

|  | Type of cancer | Method of urine analysis | Sample preparation | Number of participants | Biomarkers | Algorithm of statistical analysis | Sensitivity | Specificity | AUC |
| --- | --- | --- | --- | --- | --- | --- | --- | --- | --- |
| Nissinen (2019) | Pancreatic cancer | Field asymmetric waveform ion mobility spectrometry (FAIMS) | Urine samples were collected at the time of diagnosis and stored at -70°C. Temperature heating: 40°C | 68 pancreatic cancer,  36 acute pancreatitis,  18 chronic pancreatitis,  8 pancreatic pre-malign lesions and  52 healthy controls | - | Linear discriminant analysis (LDA), leave-one-out cross validation (LOOCV) | 79% | 79% | - |
| Daulton (2021) | Pancreatic ductal adenocarcinoma (PDAC) | Gas chromatography - ion mobility spectrometry (GC-IMS) and GC time-of-flight mass spectrometry (GC-TOF-MS) | The midstream urine samples were collected in 50 mL sterile containers and were kept at +4°C before freezing within 4 h.  Temperature and time of heating: 40°C for 10 min (GC-IMS);  40 °C for 1 h (GC-TOF-MS) | 33 healthy,  45 chronic pancreatitis  and 45 PDAC | 2,6-dimethyl-octane, nonanal, 4-ethyl-1,2-dimethyl-benzene and 2-pentanone | Random forest (RF), Gaussian process (GP), and sparse logistic regression (SLR) | GC-IMS  84%  GC-TOF-MS  72% | GC-IMS  94%  GC-TOF-MS  96% | GC-IMS  0.88  GC-TOF-MS  0.86 |
| Navaneethan (2015) | Malignant biliary strictures | Selected-ion flow-tube mass spectrometry (SIFT-MS) | Urine was obtained immediately prior to ERCP from consecutive patients with biliary strictures. Temperature heating: 40°C | 15 malignant stricture  and 39 benign strictures | 2-propranol, carbon disulfide, and trimethyl amine (TMA) | Logistic regression analysis | 93.3% | 61.5% | 0.83 |
| Khalid (2015) | Prostate cancer | Gas chromatography/mass spectrometry (GC-MS) | Stored at -20°C;  Prior to urine headspace analysis each sample was defrosted by immersing the vial in a water bath at 60°C for 30 seconds.  Temperature and time of heating: 220°C for 5 minutes  The SPME fibre was 85 μm thick and consisted of carboxen/ polydimethylsiloxane | 59 prostate cancer  and 43 cancer-free controls | 2,6-dimethyl-7-octen-2-ol, pentanal, 3-octanone, and 2-octanone | Random Forest (RF) and Linear Discriminant Analysis (LDA) | - | - | 0.76 |
| Gao (2019) | Prostate cancer | Gas chromatography-mass spectrometry (GC-MS) | Urine samples were collected and stored at -80°C prior to  VOC analysis. The  initial temperature was set at 45 °C holding for 0.5 minutes; the  temperature was increased to 300 °C at 60 °C /minute and held for  5 minutes | 55 and 53 biopsy proven PCa-positive and -negative patients | Note1 | Logistic regression models with  LASSO penalty, Firth approach, R package (OptimalCutpoints) | 96% | 80% | 0.92 |
| Tyagi (2021) | Bladder cancer (BCa) and prostate cancer (PCa) | Gas chromatography-ion mobility spectrometry (GC-IMS) and Gas chromatography time-of-flight mass spectrometry (GC-TOF-MS) | Urine samples were collected in standard universal sterile specimen containers and frozen within 2h at −80 °C. Temperature and time of heating: 40°C for 10 min (GC-IMS); 40°C for 20 min (GC-TOF-MS) | 15 BCa,  55 PCa,  and 36 NC. | Note 2 | XGBoost, logistic regression, and random forest | GC-IMS  BCa vs NC 87%  PCa vs NC  76%  GC-TOF-MS  BCa vs NC 27%  PCa vs NC  78% | GC-IMS  BCa vs NC 92%  PCa vs NC  88%  GC-TOF-MS  BCa vs NC 94%  PCa vs NC  88% | GC-IMS  BCa vs NC  0.95  PCa vs NC  0.89  GC-TOF-MS  BCa vs NC  0.81  PCa vs NC  0.94 |
| Liu (2023) | Prostate cancer | Gas chromatography-ion mobility spectrometry (GC-IMS) | Approximately 10 mL of urine samples were collected from each subject using sterile containers and stored in a −80 °C refrigerator within 3 h.  Temperature and time of heating: 100°C for 5 min | 66 PCa  and 87 NC | Furan-3-methanol, (E, E)-Octadeca-2,4-dienal, 2-Ethylhexan-1-ol, 2-Undecen-1-al | Random forests (RF), neural network (NN), support vector machines (SVM), decision trees (DT) | 91.3% | 95.7% | 0.981 |

Note 1: 4-(3,4-dihydro-2,2,4-trimethyl-2H-1-benzopyran-4-yl)-phenol, Estradiol, Ethyl à-hydroxymyristate trisiloxane, 1-(2,4-Dimethylphenyl)-3-(tetrahydrofuryl-2)propane, 2-amino-Imidazole-5-carboxylic acid, 1,1,3,3,5,5,7,7,9,9-decamethyl-pentasiloxane, 1,1,1,5,5,5-hexamethyl-3,3-bis[(trimethylsilyl)oxy]-Trisiloxane, Phthalic acid, bis(7-methyloctyl) ester, 4-Nitro-4’-chlorodiphenylsulfoxide, 1-Propylpentachlorotriphosphazene, 2,6-di-t-butyl-4-hydroxymethylene-2,3,5,6-detetrahydrocyclohexanone.

Note 2: BCa: Biphenyl, Nonanal, Tetradecane, Pentadecane, 2,6,10,14-tetramethyl-, 2-Pentanone, Undecane, 4-Heptanone, Dodecane, Hexadecane, Heptanal, Methyl Isobutyl Ketone, Naphthalene, Benzoic acid;

PCa: Toluene, Phenol, Acetic acid, 1-Hexanol, 2-ethyl-, Disulfide, dimethyl, Cyclopentanone, 2-methyl-, Pyrrole.
