# Supplementary material for: Identification of urinary volatile organic compounds as a potential non-invasive biomarker for esophageal cancer
Source: Sci Rep. 2023 Oct 30;13:18587. doi: 10.1038/s41598-023-45989-1 (PMC10616168; doi:10.1038/s41598-023-45989-1)
Supplement: Supplementary file 1 — Supplementary Information. [file 41598_2023_45989_MOESM1_ESM.zip › Supplementary files/Supplementary Table S2.docx]

**Supplementary Table S2. Thirty-seven VOC peaks were selected based on retention indices and drift times.**

| Compound | CAS# | Formula | MW | RI | Rt [sec] | Dt [a.u.] |
| --- | --- | --- | --- | --- | --- | --- |
| 2,3-Butandiol | C513859 | C4H10O2 | 90.1 | 1649.2 | 410.499 | 1.36579 |
| 2-Acetylfuran | C1192627 | C6H6O2 | 110.1 | 1640.9 | 402.241 | 1.11753 |
| 1H-Pyrrole | C109977 | C4H5N | 67.1 | 1538.1 | 312.873 | 0.97348 |
| 2,3-Diethyl-5-methylpyrazine | C18138040 | C9H14N2 | 150.2 | 1533.2 | 309.196 | 1.28871 |
| (Z)-3-Hexenyl-2-methylbutyrate | C53398859 | C11H20O2 | 184.3 | 1510 | 292.145 | 1.4569 |
| 2-methoxy-3-sec-butyl pyrazine | C24168705 | C9H14N2O | 166.2 | 1562.9 | 332.406 | 1.2845 |
| Methyl caprylate-M | C111115 | C9H18O2 | 158.2 | 1407.4 | 227.363 | 1.42163 |
| allyl isothiocyanate | C57067 | C4H5NS | 99.2 | 1362.8 | 203.918 | 1.38008 |
| Methyl caprylate-D | C111115 | C9H18O2 | 158.2 | 1413.7 | 230.912 | 1.92591 |
| 2-Octanone-M | C111137 | C8H16O | 128.2 | 1328.2 | 187.398 | 1.34279 |
| 2-Pentanone | C107879 | C5H10O | 86.1 | 987.8 | 101.321 | 1.3689 |
| 2-Butanone | C78933 | C4H8O | 72.1 | 908.5 | 94.133 | 1.25064 |
| 4-Heptanone | C123193 | C7H14O | 114.2 | 1135.2 | 124.59 | 1.59774 |
| 3-Methylbutyl 2-methylbutanoate | C27625350 | C10H20O2 | 172.3 | 1286.7 | 169.804 | 1.42266 |
| 2-Octanone-D | C111137 | C8H16O | 128.2 | 1333.2 | 189.666 | 1.76203 |
| ( E, E)-2,4-heptadienal | C4313035 | C7H10O | 110.2 | 1539.7 | 314.098 | 1.62191 |
| ( E) -2-nonenal | C18829566 | C9H16O | 140.2 | 1541.4 | 315.435 | 1.41371 |
| 2-Heptanol | C543497 | C7H16O | 116.2 | 1326.3 | 186.529 | 1.38015 |
| Dimethyl trisulfide | C3658808 | C2H6S3 | 126.3 | 1408.3 | 227.863 | 1.30062 |
| 2,6-Dimethyl-3-ethylpyrazine | C13925070 | C8H12N2 | 136.2 | 1465.8 | 262.22 | 1.21758 |
| (E)-Ethyl-2-hexenoate | C27829727 | C8H14O2 | 142.2 | 1340.6 | 193.155 | 1.32162 |
| 1 -hexanol | C111273 | C6H14O | 102.2 | 1392.6 | 219.312 | 1.32116 |
| 2-Isopropyl-3-methoxy pyrazine | C25773404 | C8H12N2O | 152.2 | 1436.9 | 244.379 | 1.24666 |
| Cyclohexanone-M | C108941 | C6H10O | 98.1 | 1295.8 | 173.127 | 1.16099 |
| Cyclohexanone-D | C108941 | C6H10O | 98.1 | 1296.3 | 173.332 | 1.4624 |
| Butyl sulfide | C544401 | C8H18S | 146.3 | 1252.2 | 157.855 | 1.29453 |
| (E)-2-Methyl-2-butenal | C497030 | C5H8O | 84.1 | 1097.3 | 115.805 | 1.34296 |
| 4-Methyl-2-pentanone | C108101 | C6H12O | 100.2 | 1019.8 | 105.146 | 1.48016 |
| 2-Methyl-butanoic acid methyl ester | C868575 | C6H12O2 | 116.2 | 1035.3 | 107.201 | 1.19254 |
| hexanal | C66251 | C6H12O | 100.2 | 1097.9 | 115.929 | 1.57568 |
| Ethyl octanoate | C106321 | C10H20O2 | 172.3 | 1524 | 302.28 | 1.4995 |
| Methyl decanoate | C110429 | C11H22O2 | 186.3 | 1796.8 | 588.696 | 1.55728 |
| 1-Octen-3-ol-D | C3391864 | C8H16O | 128.2 | 1532.9 | 308.954 | 1.59396 |
| 1-Octen-3-ol-M | C3391864 | C8H16O | 128.2 | 1533.7 | 309.546 | 1.15571 |
| Hexyl 2-methylbutanoate | C10032152 | C11H22O2 | 186.3 | 1463.1 | 260.539 | 1.52673 |
| 1-Octen-3-one | C4312996 | C8H14O | 126.2 | 1322.8 | 184.919 | 1.27339 |
| (2,6)-dimethylpyrazine | C108509 | C6H8N2 | 108.1 | 1342.4 | 193.994 | 1.13432 |
