# Supplementary material for: Identification of urinary volatile organic compounds as a potential non-invasive biomarker for esophageal cancer
Source: Sci Rep. 2023 Oct 30;13:18587. doi: 10.1038/s41598-023-45989-1 (PMC10616168; doi:10.1038/s41598-023-45989-1)
Supplement: Supplementary file 1 — Supplementary Information. [file 41598_2023_45989_MOESM1_ESM.zip › Supplementary files/Supplementary Table S4.docx]

**Supplementary Table S4. Identification of HCs and stage I-IV EC using 8-VOCs model.**

| Stage | Accuracy | Precision | Recall | F1 | Specificity | AUC |
| --- | --- | --- | --- | --- | --- | --- |
| HC vs I-stage | 0.977 | 0.889 | 1.000 | 0.941 | 0.972 | 0.986 |
| HC vs II-stage | 0.973 | 0.927 | 0.974 | 0.950 | 0.972 | 0.973 |
| HC vs III-stage | 0.947 | 0.930 | 0.889 | 0.909 | 0.972 | 0.930 |
| HC vs IV-stage | 0.976 | 0.850 | 1.000 | 0.919 | 0.972 | 0.986 |

Note: Recall is equivalent to sensitivity.
