# Supplementary material for: Identification of urinary volatile organic compounds as a potential non-invasive biomarker for esophageal cancer
Source: Sci Rep. 2023 Oct 30;13:18587. doi: 10.1038/s41598-023-45989-1 (PMC10616168; doi:10.1038/s41598-023-45989-1)
Supplement: Supplementary file 1 — Supplementary Information. [file 41598_2023_45989_MOESM1_ESM.zip › Supplementary files/Supplementary Table S5.docx]

| **Supplementary Table S5. Identification of HCs and Stage I-IV EC based on VOCs.** | | | | |
| --- | --- | --- | --- | --- |
| **VOC molecular** | **HC vs I-stage** | **HC vs II-stage** | **HC vs III-stage** | **HC vs IV-stage** |
| **2,3-Butandiol** | **<0.001** | **<0.001** | **<0.001** | **<0.001** |
| **2-Acetylfuran** | **<0.001** | **<0.001** | **<0.001** | 0.301 |
| **Dimethyl trisulfide** | **<0.001** | **0.001** | **0.003** | **0.046** |
| **(E)-Ethyl-2-hexenoate** | **0.009** | **<0.001** | **<0.001** | 0.227 |
| **2-Isopropyl-3-methoxy pyrazine** | **0.001** | **<0.001** | **0.020** | 0.870 |
| **Cyclohexanone-D** | **<0.001** | **<0.001** | **<0.001** | **<0.001** |
| **2-Methyl-butanoic acid methyl ester** | 0.070 | 0.069 | **0.005** | **0.006** |
| **Methyl decanoate** | **<0.001** | **<0.001** | **<0.001** | **<0.001** |
| Data represent P-values. |  |  |  |  |
| *P* values are from Mann-Whitney U test. | |  |  |  |
