# Supplementary figures and images for: Identification of urinary volatile organic compounds as a potential non-invasive biomarker for esophageal cancer
Source: Sci Rep. 2023 Oct 30;13:18587. doi: 10.1038/s41598-023-45989-1 (PMC10616168; doi:10.1038/s41598-023-45989-1)

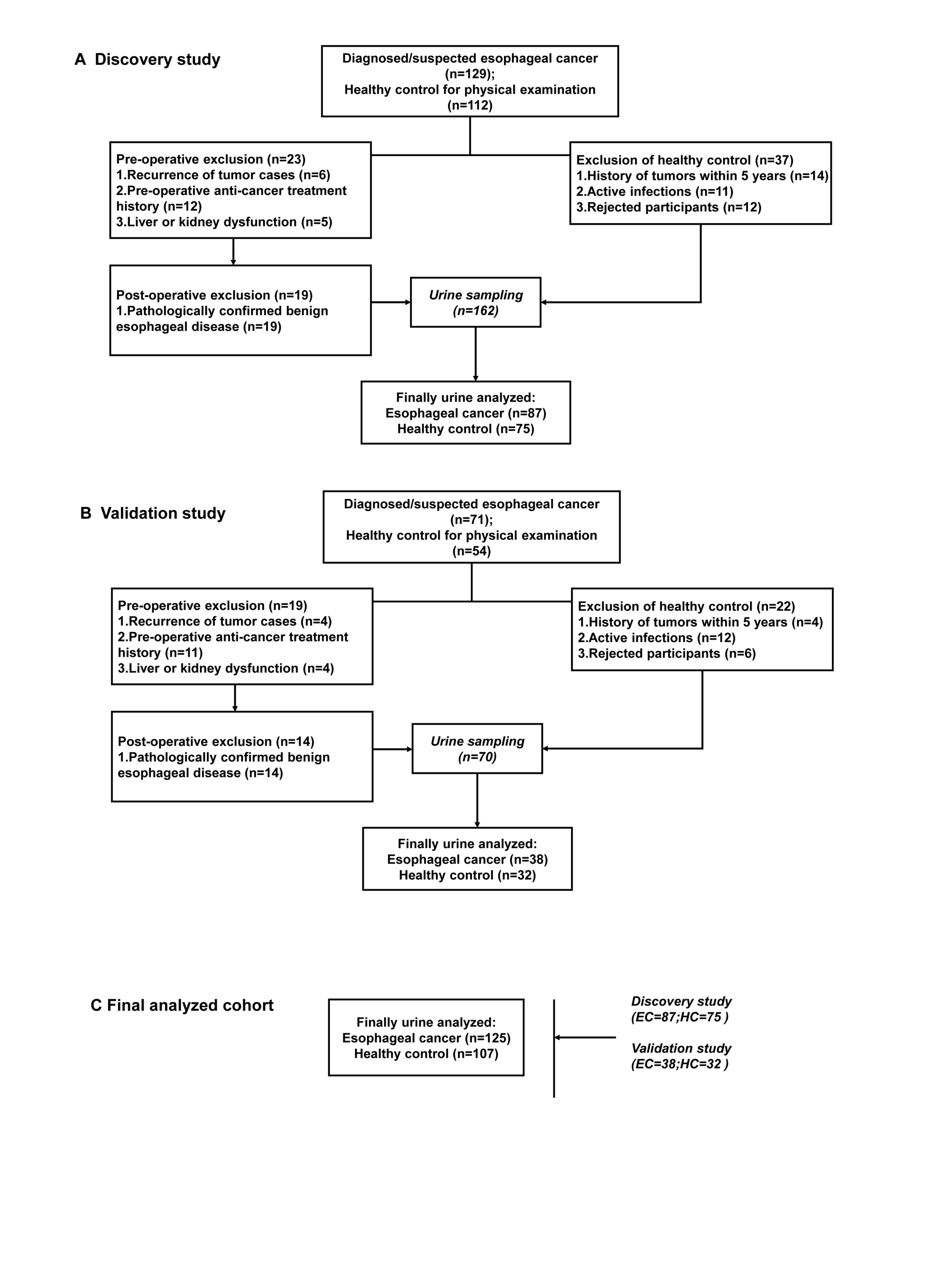

Supplement: Supplementary file 1 — Supplementary Information. [file 41598_2023_45989_MOESM1_ESM.zip › Supplementary files/Supplementary Figure S1.tif]

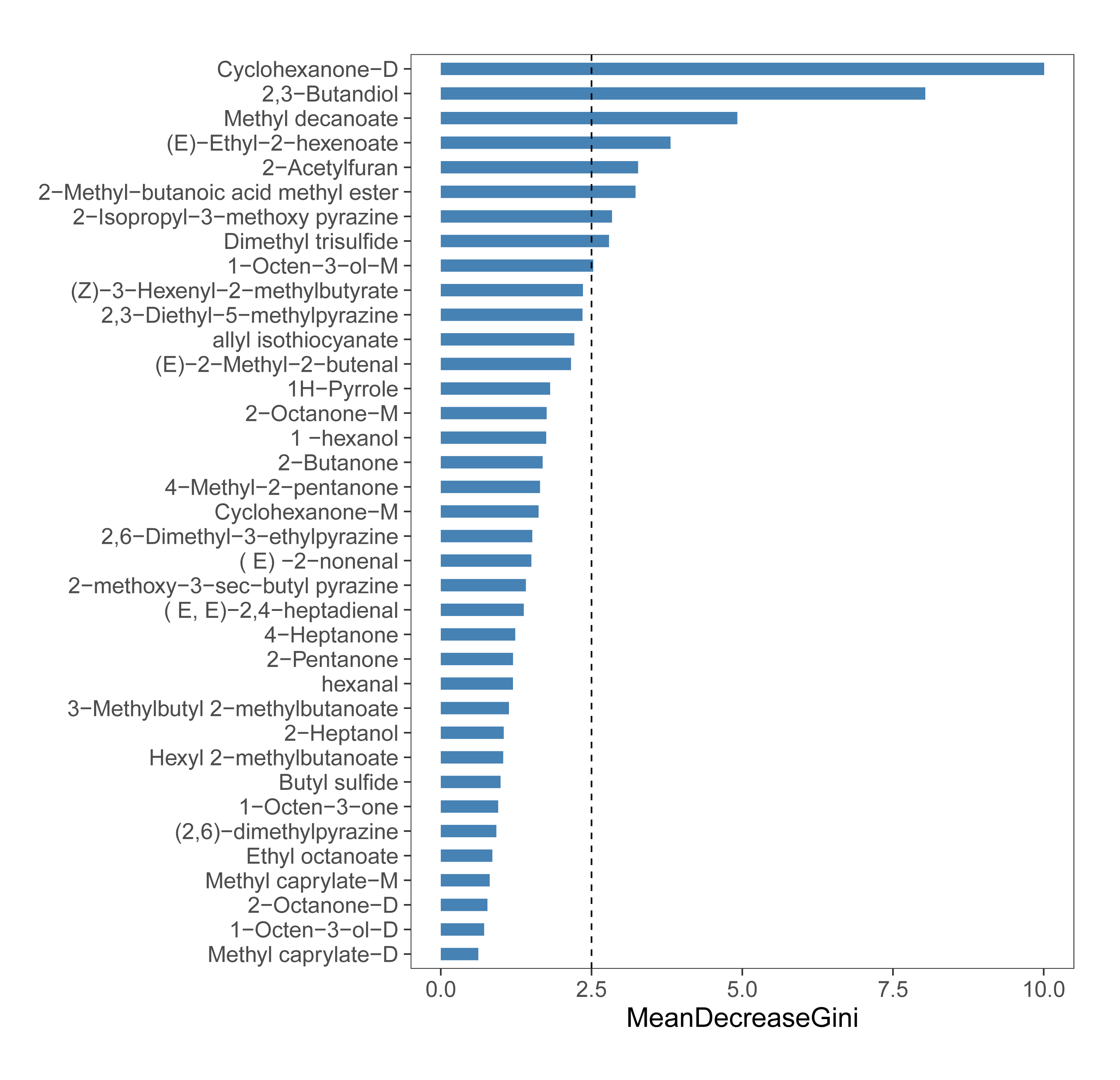

Supplement: Supplementary file 1 — Supplementary Information. [file 41598_2023_45989_MOESM1_ESM.zip › Supplementary files/Supplementary Figure S2.tif]

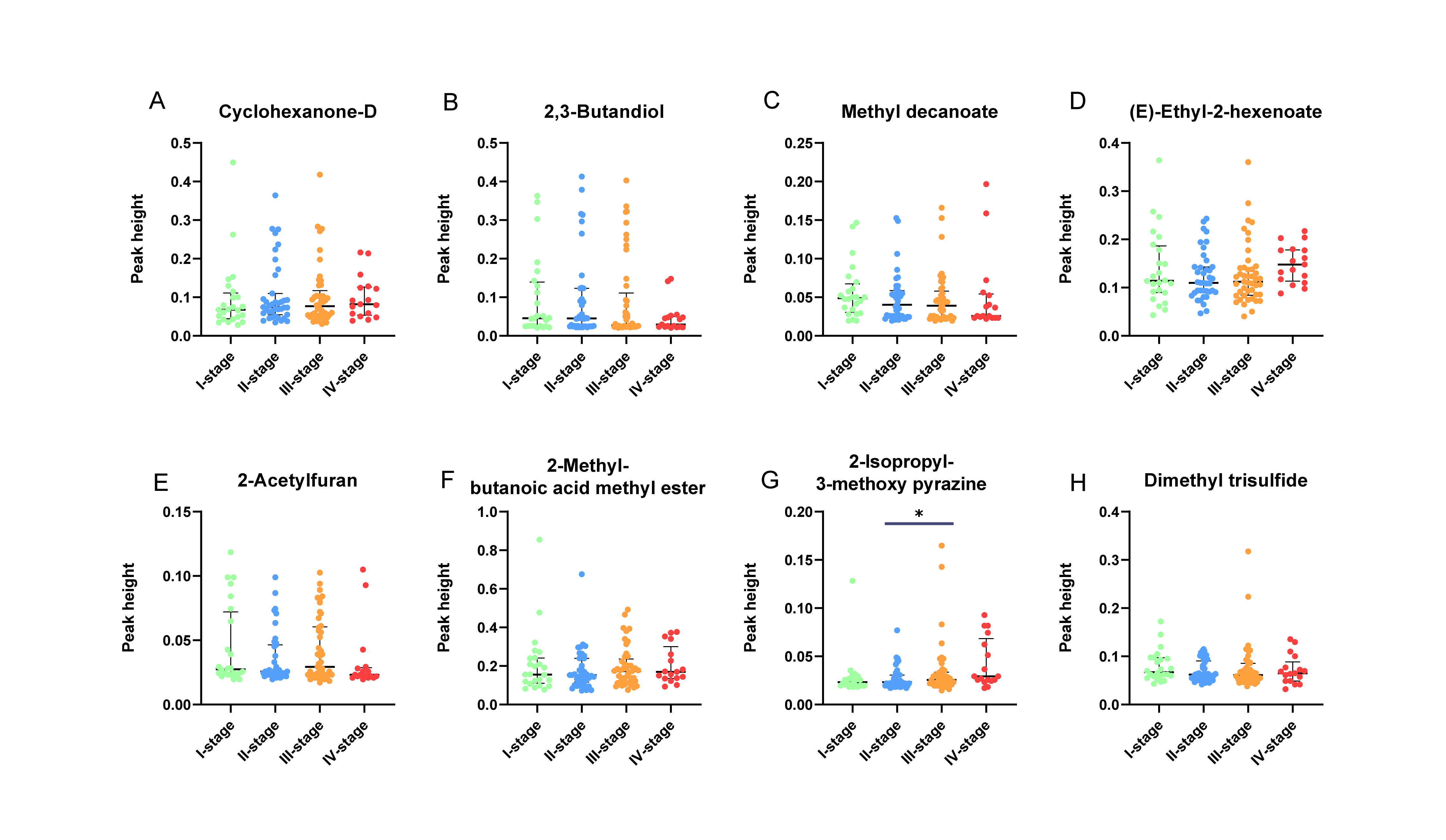

Supplement: Supplementary file 1 — Supplementary Information. [file 41598_2023_45989_MOESM1_ESM.zip › Supplementary files/Supplementary Figure S3.tif]

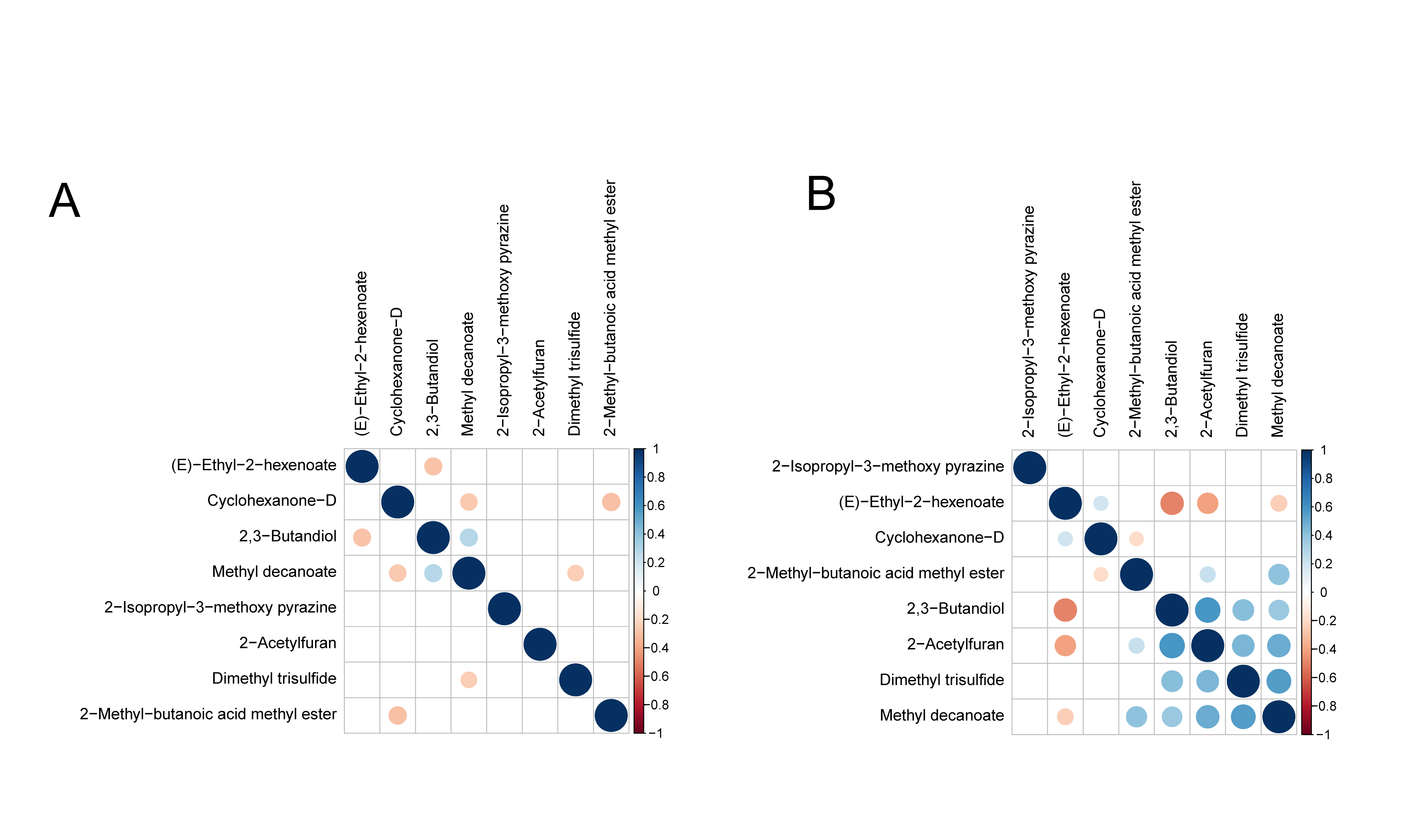

Supplement: Supplementary file 1 — Supplementary Information. [file 41598_2023_45989_MOESM1_ESM.zip › Supplementary files/Supplementary Figure S4.tif]
